# The Combination of *α*-Tocopheryl Succinate and Sodium Selenite on Breast Cancer: A Merit or a Demerit?

**DOI:** 10.1155/2016/4741694

**Published:** 2016-03-29

**Authors:** Doaa M. Badr, Hafez F. Hafez, Azza M. Agha, Samia A. Shouman

**Affiliations:** ^1^Pharmacology Unit, Cancer Biology Department, National Cancer Institute, Cairo University, Cairo 11796, Egypt; ^2^Pharmacology and Toxicology Department, Faculty of Pharmacy, Cairo University, Cairo 11796, Egypt

## Abstract

*α*-Tocopheryl succinate (*α*-TOS), a mitochondria-targeting agent, induces apoptosis in malignant cells in vitro and in vivo. Selenite is a nutritional supplement that has been shown to stimulate apoptosis in cancer cells. This study was designed to investigate the cytotoxic effect of combined treatment of *α*-TOS and sodium selenite (SSe) in vitro and in vivo and to explore their effect on apoptosis and autophagy in breast cancer. The type of interaction between *α*-TOS and SSe was evaluated and levels of oxidative stress and apoptotic and autophagic markers were determined. SSe alone showed varying degrees of cytotoxicity on all the tested cell lines. Its combination with *α*-TOS was antagonistic in vitro in MCF7 and in vivo in mice bearing Ehrlich tumor compared to *α*-TOS-treated one. Combination of TOS with 2 *μ*M of SSe increased the level of glutathione without changes in antiapoptotic markers Bcl-2 and Mcl-1 at 16 and 48 hrs. SSe decreased caspase 3 activity and protein level of caspases 7 and 9, while it increased autophagic markers beclin-1 and LC3B protein levels of MCF7 cells treated with *α*-TOS. In conclusion, SSe antagonizes *α*-TOS-induced apoptosis via inhibition of oxidative stress and promoting prosurvival machinery of autophagy.

## 1. Introduction

Breast cancer is the second most common cancer in the world and the most frequent cancer among women with an estimated 1.67 million new cancer cases diagnosed in 2012. It is the most common cancer in women both in more and in less developed regions [1]. Despite modern approach in improving patient compliance by combating breast cancer with more selective and less toxic drugs, treatment strategies remain a challenge. *α*-Tocopheryl succinate (*α*-TOS) is a provitamin known to reduce the growth of various cancer cell lines, by virtue of its prooxidant effect, including breast, prostate, pancreatic, and melanoma cancers, while shielding normal cells [2–5]. In addition, in vivo, *α*-TOS causes suppression of chemically induced forestomach cancer [6] and inhibits the growth of several inoculated cancer cells [7, 8].

Modulation of signaling pathways by micronutrients is a promising strategy for cancer prevention and treatment. It was demonstrated that combination of polar carotenoids is more effective against the growth of tumor cells in vitro than the individual agents [9]. Selenium is an essential dietary micronutrient for all mammalian species. Selenium-replete diets are thought to result in maximal expression of selenoproteins [10], a family of proteins whose functions include antioxidant activities and maintaining the intracellular redox state [11]. Sodium selenite (SSe) exhibits greater toxicity towards malignant than benign cells [12] and inhibits the development of mammary tumors in a rat model [11]. Selenite-mediated generation of the superoxide radical anion (O_2_^∙^) is thought to cause oxidative stress, leading to cellular damage and death. The O_2_^∙^ observed in selenite-treated cells may originate from mitochondria [13]. SSe was reported to induce DNA damage, particularly DNA strand breaks. Furthermore, it may sensitize malignant cells to apoptosis induced by other antineoplastic treatment modalities, thereby improving the efficacy and outcome of potential antineoplastic therapy [14]. Hence the combination of *α*-TOS with SSe may have superior antitumor activity to *α*-TOS alone. Therefore, the aim of this study was to test the combined antitumor activity of *α*-TOS and SSe via assessing some apoptotic and autophagic markers.

## 2. Materials and Methods

### 2.1. Human Cancer Cell Lines

In this study, a panel of the available cell lines were tested for their chemosensitivity to either *α*-TOS or SSe. Two concentrations (nutritional 2 *μ*M and supranutritional 10 *μ*M) of SSe were used in combination with different concentrations of *α*-TOS, and type of drug interaction was evaluated for all the tested cell lines. The most sensitive cell line to the treatment regimen was selected and subjected to further investigations to explore the mechanism of this interaction. Breast adenocarcinoma MCF7, cervical adenocarcinoma HeLa, lung carcinoma A549, mammary gland ductal carcinoma T47D, prostate adenocarcinoma PC3, hepatocellular carcinoma HepG2, and colorectal carcinoma HCT-116 cell lines were obtained from American Type Culture Collection (ATCC) (Rockville, MD, USA) and maintained at the National Cancer Institute in RPMI-1640 medium containing 10% fetal bovine serum and 1% penicillin-streptomycin and routinely incubated with 5% CO_2_ in a humidified atmosphere at 37°C.

### 2.2. Cytotoxicity Assay

The cytotoxicity assay was carried according to the method described by Skehan et al. [15]. Cells were seeded in a 96-well plate at density of 3 × 10^3^ cells/well and were incubated overnight at 37°C in humidified 5% CO_2_ incubator. Cells were treated with 20–100 *μ*M *α*-TOS (Sigma Aldrich, USA) and with 2–10 *μ*M SSe (Sigma Aldrich, USA) for 48 and/or 72 hours. Control cells were treated with the RPMI-1640 medium (Biowest, France) containing 0.1% DMSO. A combination regimen was designed using the following regimens:

- *1st Combination Regimen*. Fixed 2 *μ*M SSe with 20–100 *μ*M *α*-TOS concentrations.
- *2nd Combination Regimen*. Fixed 10 *μ*M SSe with 20–100 *μ*M *α*-TOS concentrations.

After the desired time intervals, the cells were fixed with 20% trichloroacetic acid (Sigma Aldrich, USA), washed, and stained with 0.4% sulforhodamine-B dye (Sigma Aldrich, USA). The produced color was measured spectrophotometrically at 575 nm using ELISA plate reader (Tecan Sunrise*™*, Germany).

### 2.3. Evaluation of Drug Interaction

The degree of interaction between the two drugs was calculated using the combination index, according to the isobologram equation [16]: CI = *d*1/*D*1 + *d*2/*D*2, where *d*1 and *d*2 represent the concentrations of *α*-TOS and SSe that, when given in combination, produce a specific response, and *D*1 and *D*2 represent the concentrations of *α*-TOS and SSe that, when given individually, produce the same effect. When combination index values are less than 1 (CI < 1) they indicate synergism, while CI = 1 represents additivity and CI > 1 indicates antagonism.

### 2.4. Determination of *α*-TOS Uptake by MCF7 Cells

MCF7 cells 10 × 10^3^/well were seeded in colorless RPMI-1640 medium and left for 24 hours. The plate was divided into 2 groups as follows:

- Group I: treated with 60 *μ*M *α*-TOS.
- Group II: treated with 60 *μ*M *α*-TOS and 2 *μ*M SSe.

The medium was then aspirated after 0-, 2-, 4-, 6-, and 24-hour intervals and centrifuged and the supernatant was stored at −20°C till HPLC assay.

#### 2.4.1. Sample Extraction and Preparation

100 *μ*L of the medium was mixed thoroughly with 1.4 mL acetonitrile (Alliance Bio, USA) and centrifuged at 10 000 rpm for 10 minutes at 4°C. Twenty microliters of the resulting supernatant was then injected into an HPLC system consisting of 520 pump gradient, 560 autosampler, and 535 spectrophotometric detector (Bio-Tek, Italy). The analytical column used was Equisil ODS (250 mm × 4.6 mm ID, 10 *μ*m). The mobile phase consisted of acetonitrile and water (90 : 10 v/v) and flow rate of 2 mL/min, with ultraviolet detection at 205 nm [17]. Results are expressed as *μ*M *α*-TOS after calibration with standard curve (10–60 *μ*M) for *α*-TOS.

### 2.5. Determination of Total Lipid Peroxides Content (Measured as Malondialdehyde (MDA))

Lipid peroxidation products were quantified by measuring MDA level [18]. Treated and control cells were mixed well with 20% (w/v) trichloroacetic acid (TCA) containing 0.8% (w/v) thiobarbituric acid (TBA) (Sigma Aldrich, USA), incubated in a boiling water bath for 1 hour. The absorbance of the supernatant was determined at 535 nm using a spectrophotometer (Spectronic, Milton Roy Co., USA). The concentrations were calculated using MDA standard calibration curve by preparing serial dilutions of 1,1,3,3-tetraethoxypropane (Sigma Aldrich, USA).

### 2.6. Determination of Non-Protein SH (GSH) Content

Reduced glutathione was determined adopting Ellman's method [19]. MCF7 cells were harvested as previously mentioned, protein was precipitated with trichloroacetic acid, and Ellman's reagent [5,5′-dithiobis-(2-nitrobenzoic acid)] (DTNB) (Sigma Aldrich, USA) was added to clear supernatant. The absorbance was read at 405 nm and total SH was calculated as *μ*M of GSH/mg protein.

### 2.7. Western Blot

The method was carried out according to Maniatis et al. [20]. Treated and control cells were incubated with lysis buffer [150 mM NaCl (Riedel-deHaën, Germany), 10 mM Trizma (MP Biochemical, France), 0.2% triton X-100 (MP Biochemical, France), 0.3% NP-40 (Fluka BioChemika, Switzerland), and 0.2 mM sodium orthovanadate (Sigma Aldrich, USA)] for 30 minutes on ice. Cells were homogenized by repeated sonication and vortex for 30 seconds and centrifuged at 14000 g for 15 minutes at 4°C. The supernatant was collected and the protein concentration was determined. Protein was separated by 10% SDS-PAGE and electroblotted onto PVDF membrane with primary mouse anti-human caspase 9 mAB (1 : 1000) (eBioscience, Austria), caspase 7 mAB (1 : 500) (Novus Biologicals, USA), Bcl-2 mAB (1 : 2000) (Sigma Aldrich, USA), Mcl-1 mAB (1 : 500) (R&D, USA), beclin-1 mAbs (1 : 500) (R&D, USA), and rabbit anti-human LC3B oligoclonal AB (1 : 500) (Invitrogen, USA), *β*-actin mAb (1 : 1000) (R&D, USA). The protein bands were visualized using Amersham*™* ECL Western Blotting Detection Reagents on X-ray film (Fujifilm, Tokyo, Japan) after incubation of the membrane with the appropriate secondary goat anti-mouse IgG or secondary goat anti-rabbit IgG antibodies (Sigma Aldrich, USA). Images were acquired with a scanner and analyzed with Scion Image Beta 4.0.2 (Scion Co., Frederick, MD, USA) software.

### 2.8. Determination of Enzymatic Activity of Caspase 3 in Cell Lysate

Caspase 3 activity was determined in cell lysate using caspase 3 activity colorimetric assay kit purchased from R&D, USA, according to the method described by Casciola-Rosen et al. [21]. Cells were harvested after exposure to different treatment regimens and caspase 3 activity was measured in accordance with the manufacturer's instructions. The results were expressed as fold increase in caspase activity of apoptotic cells over that of nonapoptotic cells.

### 2.9. Detection of Acidic Vesicular Organelles

Autophagy was detected using the lysosomotropic agent, acridine orange (Sigma Aldrich, USA) [22]. Treated and nontreated MCF7 cells were incubated with medium containing 1 *μ*g/mL acridine orange for 10 minutes. The micrographs were taken using an inverted fluorescent microscope equipped with digital camera (NIKON, Japan) and supplied with blue (excitation BP 450–490) and green filters (excitation BP 510–550).

### 2.10. Determination of Human VEGF in Culture Medium by ELISA Kit

The culture medium of treated and control cells was aspirated and centrifuged at 10 000 rpm at 4°C for 10 minutes and the resultant supernatant was used for determination of VEGF by an ELISA kit (RayBio, USA) in accordance with the manufacturer's instructions [23]. The amount of VEGF was expressed as pg/mL.

### 2.11. Protein Assay

Protein content was determined in whole cell lysate according to the method described by Bradford [24] following the manufacturer's instructions of protein assay kit (Pierce Biotechnology, USA). Absorption was read at 595 nm with a spectrophotometer.

### 2.12. Assessment of the Antitumor Activities of *α*-TOS, SSe, and Their Combination

To explore the effect of this combination in vivo, we used the available model of animal bearing tumor [mice bearing Ehrlich ascites carcinoma (EAC)]. Female mice were transplanted subcutaneously in the right thigh with EAC cells (2 × 10^6^) till reaching a palpable tumor mass (100 mm^3^). The mice were divided into 4 groups (each group contained six mice) and injected intraperitoneally (i.p.) twice, every third day as follows:

 Gr 1: injected with DMSO (0.1 mL/20 gm) and used as control.
 Gr 2: injected with *α*-TOS (150 mg/kg) with a total dose of 300 mg/kg.
 Gr 3: injected with SSe (0.5 mg/kg) with a total dose of 1 mg/kg.
 Gr 4: injected with a simultaneous combination of *α*-TOS and SSe at the aforementioned doses.

The change in tumor volume was measured every other day using a caliper and calculated according to the following formula [25]:

(1) $$\begin{array}{c} Tumor\;\;volume\;\;\left(\;{mm}^{3}\;\right)=0.52\times A^{2}\times B^{2}, \end{array}$$

where *A*, *B* refer to the minor and major tumor axis, respectively.

### 2.13. Assessment of the Oncolytic Activities of *α*-TOS, SSe, and Their Combination

Female mice were injected i.p. with EAC. Twenty-four hours after cell inoculation, the mice were divided into 4 groups (each group contained ten mice), injected i.p. on 2 consecutive days with the aforementioned treatment regimens. The animals were observed daily and the percent survival, mean survival time (days), percent change in animal and body weight, and percent change in life span (% CLS) were calculated: % CLS = (*T* − *C*/*C*) × 100, where

 
*T* =* average life span of treated mice*,
 
*C* =* average life span of control mice*.

### 2.14. Statistical Analysis

Unpaired *t*-test was used to compare two different treatment groups. Multiple comparisons were carried out using one-way analysis of variance (ANOVA) followed by Tukey-Kramer test for post hoc analysis. Statistical significance was acceptable at a level of *P* value < 0.05. Graphs were performed using Prizm software program (GraphPad Prism software, version 5).

## 3. Results

### 3.1. SSe Antagonizes *α*-TOS Cytotoxicity in All the Tested Cell Lines

Results of the present study revealed that all the tested cell lines were resistant to concentrations of SSe that were used in this study (2–10 *μ*M), IC_50_ > 10 *μ*M except MCF7 which showed IC_50_ at 5.45 *μ*M, Table 1. Moreover, when the cell lines were exposed to 20–100 *μ*M of *α*TOS IC_50_ was >100 *μ*M in Hela and T47D, and the cell lines IC_50_ ranged from 77 to 100, while in MCF7 it was 57.5 *μ*M after 48 hours; data are shown in Supplementary Material (see Supplementary Material available online at http://dx.doi.org/10.1155/2016/4741694). Treatment of all the cell lines, with combination of either 2 or 10 *μ*M of SSe and different concentrations of *α*TOS, resulted in antagonistic effect, Table 1. Therefore MCF7 cell line was chosen, as it is the most sensitive cell line that showed growth inhibition to each drug alone.

### 3.2. SSe Antagonizes *α*-TOS Cytotoxicity in MCF7

**Figure 1:**
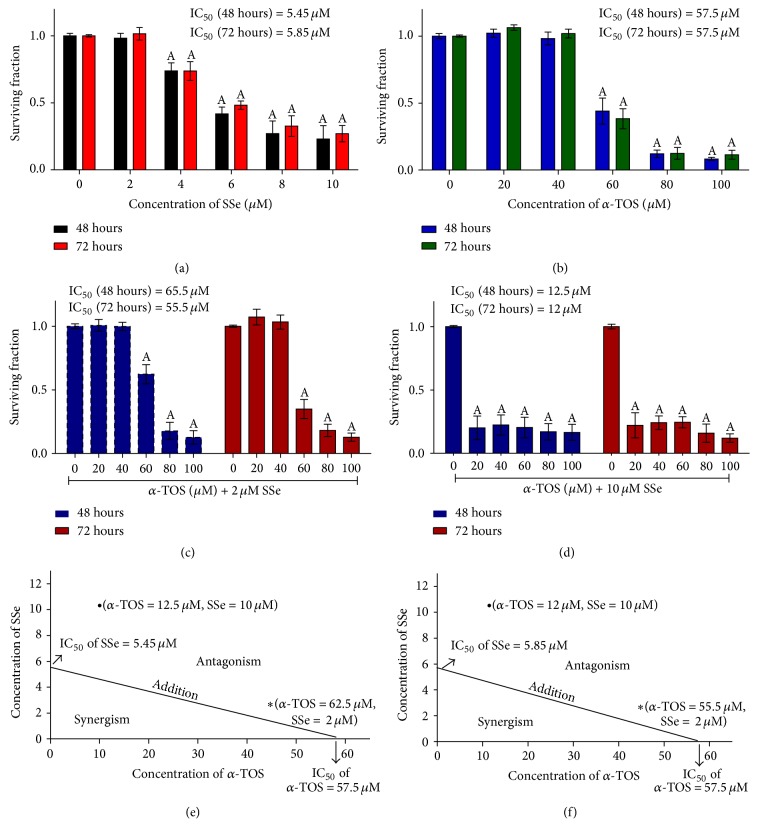
Surviving fraction of MCF7 cells treated with (a) SSe (2–10 *μ*M) and (b) *α*-TOS (20–100 *μ*M) and its combination with (c) 2 *μ*M and (d) 10 *μ*M SSe and their combination indices are represented in the isobologram after (e) 48 and (f) 72 hours. The results are expressed as mean ± SD of 5 independent experiments performed in triplicate. Statistical significance of results was analyzed by one-way ANOVA using Tukey's multiple comparison test. “A” significantly different from its respective control at *P* < 0.05.

Treatment of MCF7 cells with *α*-TOS or SSe alone resulted in decrease in cellular growth after 48 and 72 hours, Figures 1(a) and 1(b). The effect of combination of *α*-TOS with either 2 or 10 *μ*M SSe was antagonistic on MCF7 cell line after 48 and 72 hours (Figures 1(c), 1(d), 1(e), and 1(f)). Therefore, we used the nontoxic concentration of SSe (2 *μ*M) to study the mechanism of this antagonistic effect between *α*-TOS and SSe on MCF7 cells following 16 and 48 hours.

### 3.3. SSe Does Not Affect Cellular Level of *α*-TOS

**Figure 2:**
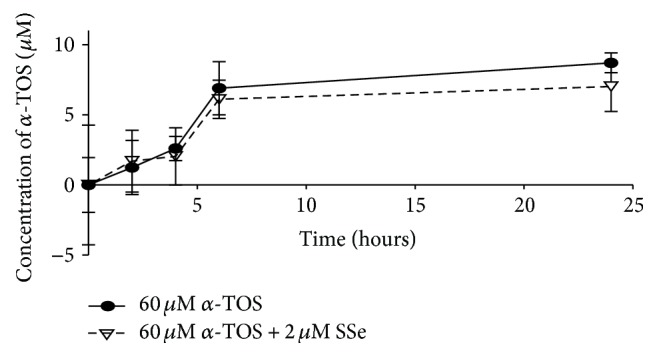
Effect of SSe on cellular uptake of *α*-TOS from the culture medium after different time intervals (0, 2, 4, 6, and 24 hours). Results are expressed as means ± SD of 3 independent experiments. Results were found nonsignificantly different at *P* < 0.05 using unpaired *t*-test.

SSe did not affect significantly the cellular uptake of *α*-TOS at any point of the studied time (Figure 2).

### 3.4. SSe, at Nutritional Concentration, Acts as an Antioxidant While, at Super Nutrition Concentration, It Acts as Prooxidant

**Figure 3:**
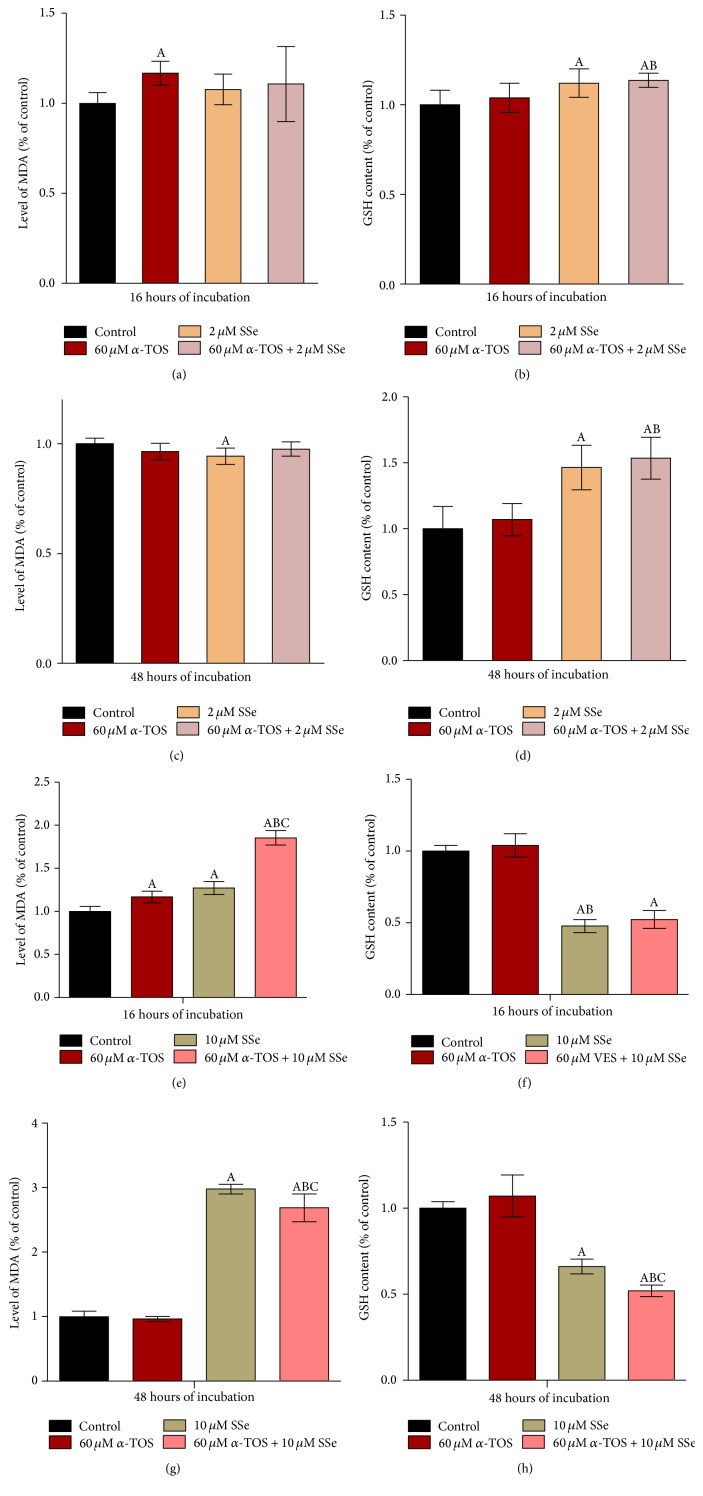
Combined effect of 60 *μ*M *α*-TOS and 2 *μ*M SSe on lipid peroxidation after (a) 16 hours and (c) 48 hours and on reduced glutathione levels after (b) 16 hours and (d) 48 hours. Combined effect of 60 *μ*M *α*-TOS and 10 *μ*M SSe on lipid peroxidation after (e) 16 hours and (g) 48 hours and on reduced glutathione levels after (f) 16 hours and (h) 48 hours in MCF7 cells. Results of MDA and rGSH are expressed as means ± SD of 5 independent experiments (*n* = 15 for MDA and *n* = 12 for rGSH). Statistical significance of results was analyzed by one-way ANOVA using Tukey's multiple comparison test. “A” significantly different from the respective control at *P* < 0.05; “B” significantly different from respective *α*-TOS at *P* < 0.05; “C” significantly different from respective SSe at *P* < 0.05.

*α*-TOS produced a significant increase in MDA level after 16 hours, followed by a recovery to control value after 48 hours. On the other hand, low concentration of SSe (2 *μ*M) significantly decreased MDA levels after 48 hours, whereas higher concentration of SSe (10 *μ*M) significantly increased MDA level after both time intervals (Figures 3(a), 3(c), 3(e), and 3(g)). Regarding GSH level, it was shown that SSe (2 *μ*M) alone produced significant increase in GSH by 12% and 46% at 16 and 48 hrs, while SSe at 10 *μ*M concentration significantly reduced GSH level to 47.7% and 66% at 16 and 48 hrs, respectively. In addition, GSH level was significantly increased following exposure of cells to the combination of *α*-TOS and 2 *μ*M SSe (Figures 3(b) and 3(d)), while combination with 10 *μ*M SSe significantly decreased GSH level (Figures 3(f) and 3(h)).

### 3.5. *α*-TOS, SSe, and Their Combinations Do Not Affect Bcl-2 and Mcl-1 Protein Levels

**Figure 4:**
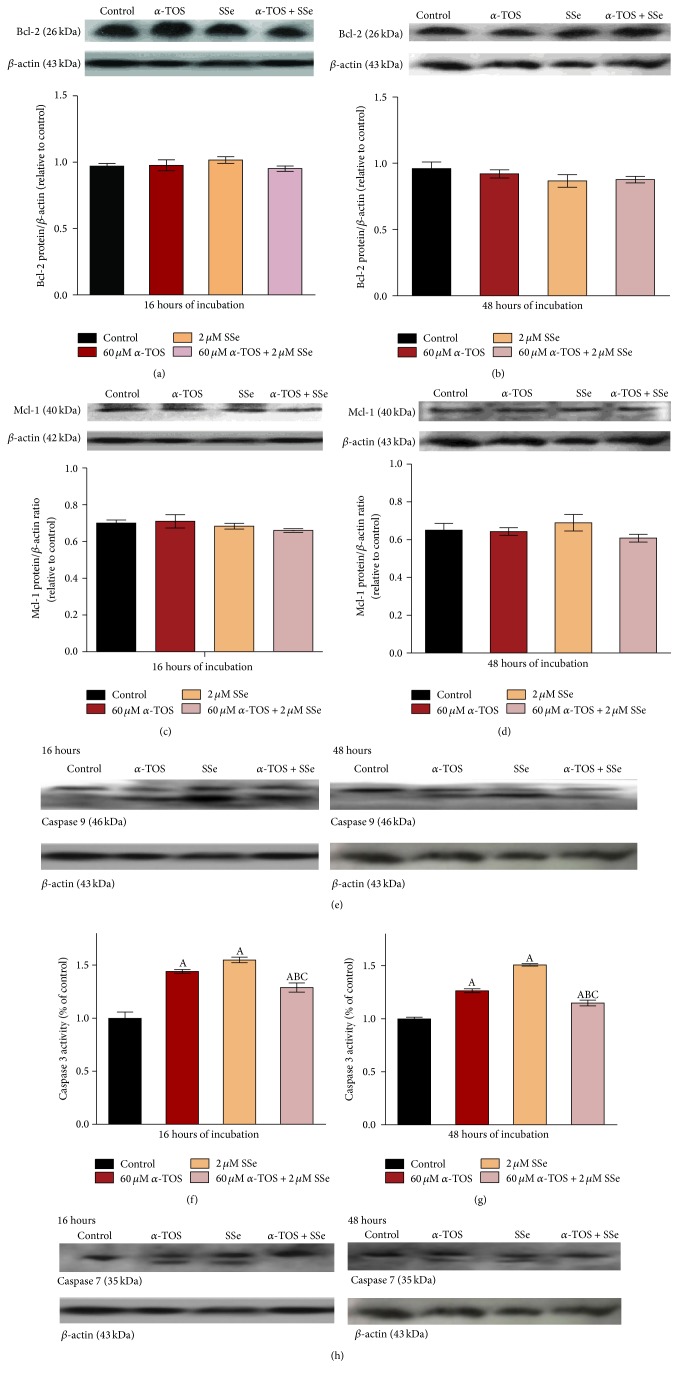
Effect of 60 *μ*M *α*-TOS, 2 *μ*M SSe, and their combination on Bcl-2 protein levels after (a) 16 and (b) 48 hours, on Mcl-1 protein levels after (c) 16 and (d) 48 hours, on (e) caspase 9 and (h) caspase 7 activation after 16 and 48 hours using western blot technique, and on enzymatic activity of the caspase 3 class of proteases in cell lysate of MCF7 cells after (f) 16 and (g) 48 hours of exposure using ELISA technique. Western blot results were expressed as means ± SD of 3 independent experiments (*n* = 3). ELISA results are expressed as means ± SD of 2 replicate experiments (*n* = 4). Statistical significance of results was analyzed by one-way ANOVA using Tukey's multiple comparison test. “A” significantly different from the respective control at *P* < 0.05; “B” significantly different from respective *α*-TOS at *P* < 0.05; “C” significantly different from respective SSe at *P* < 0.05.

Using scion image to precisely measure the protein level, it was found that the antiapoptotic protein levels of Bcl-2 or Mcl-1 did not change with any treatment regimen for both time intervals used (Figures 4(a), 4(b), 4(c), and 4(d)).

### 3.6. Either *α*-TOS or SSe Individually Increases Caspases, While Their Combinations Decrease Them

Both *α*-TOS and SSe resulted in activation and cleavage of caspase 9 and caspase 7 proteins as well as a significant increase in caspase 3 activity, following incubation for 16 and 48 hours compared to the control. Interestingly, the combination resulted in a significant inhibition of the activation of caspase 7 and activity of caspase 3 leading to inhibition of apoptosis (Figures 4(e), 4(f), 4(g), and 4(h)).

### 3.7. *α*-TOS, SSe, and Their Combinations Increase the Expression of Autophagic Proteins

**Figure 5:**
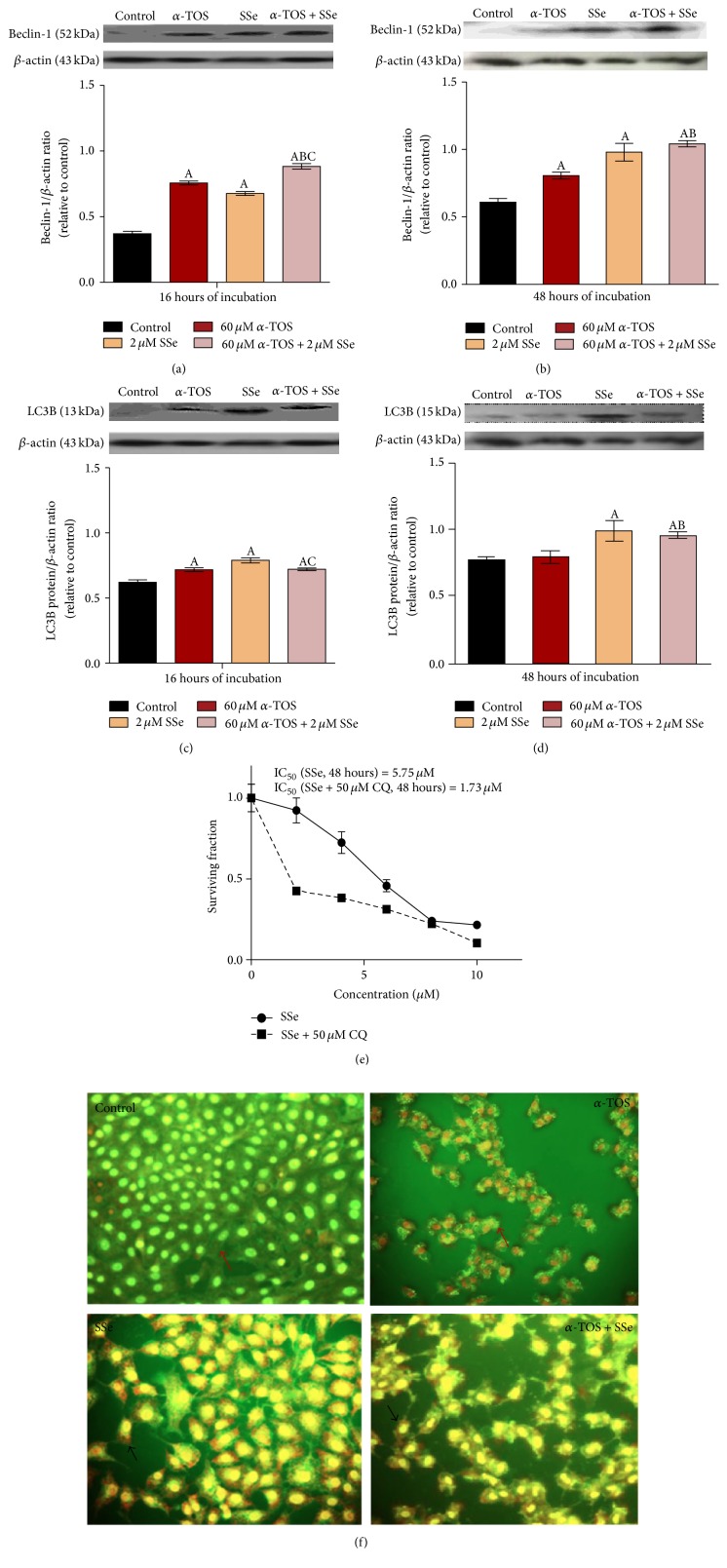
Effect of 60 *μ*M *α*-TOS, 2 *μ*M SSe, and their combination on beclin-1 protein level following (a) 16 hours and (b) 48 hours and on LC3B protein level following (c) 16 hours and (d) 48 hours of exposure in MCF7 cells using western blot technique and (e) effect of CQ (50 *μ*M) on IC_50_ of SSe after 48 hours. Acridine orange stained MCF7 cells after exposure to 60 *μ*M *α*-TOS, 2 *μ*M SSe, and their combination after (f) 48 hours [original magnification (objective lens (20x))]. Cells with acidic vesicular organelles (AVOs) can be visualized by their orange fluorescence (pointed by black arrows) in the cytoplasm, whereas cells without AVOs are marked by red arrows. Western blot results are expressed as means ± SD of 3 independent experiments (*n* = 3). Statistical significance of results was analyzed by one-way ANOVA using Tukey's multiple comparison test. “A” significantly different from the respective control at *P* < 0.05; “B” significantly different from respective *α*-TOS at *P* < 0.05; “C” significantly different from respective SSe at *P* < 0.05.

SSe either alone or combined with *α*-TOS induced autophagy after both 16 and 48 hours of exposure. This was shown by an increase in beclin-1 and LC3B protein levels after both time intervals. On the other hand, *α*-TOS induced an early autophagy process, where the levels of both proteins were increased after 16 hours, but there was a recovery in LC3B level within 48 hours (Figures 5(a), 5(b), 5(c), and 5(d)). To determine the interplay between autophagy induction by SSe and apoptosis, cells were pretreated with chloroquine (CQ), an inhibitor of autophagy, prior to treatment with various concentrations (2–10 *μ*M) of SSe for 48 hours. The IC_50_ was shifted to be 1.73 *μ*M, indicating that inhibition of autophagy increased the apoptotic effect (Figure 5(e)). This was further elucidated by AO staining of cytoplasmic AVOs, which were detected after 48 hours in the cytoplasm of SSe and combination-treated cells but not in the *α*-TOS-treated cells (Figure 5(f)).

### 3.8. *α*-TOS and Its Combination with SSe Inhibit the Release of Human VEGF

**Figure 6:**
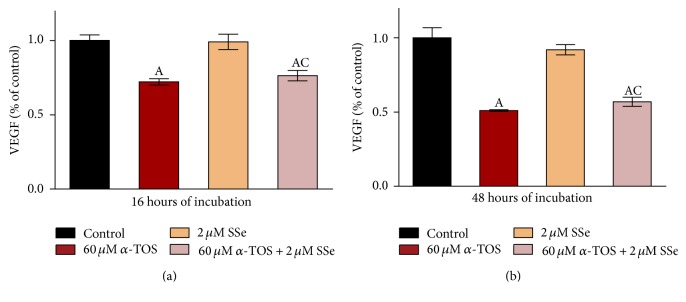
Combined effect of 60 *μ*M *α*-TOS and 2 *μ*M SSe on VEGF secretion by MCF7 cells following (a) 16 hours and (b) 48 hours of incubation. Results are expressed as means ± SD of 2 replicate experiments (*n* = 4). Statistical significance of results was analyzed by one-way ANOVA using Tukey's multiple comparison test. “A” significantly different from the respective control at *P* < 0.05; “B” significantly different from respective *α*-TOS at *P* < 0.05; “C” significantly different from respective SSe at *P* < 0.05.

The results revealed that secretion of VEGF into culture medium was significantly decreased in *α*-TOS-treated group after both time intervals, whereas SSe exposure for either time interval showed no significant change in its level as compared to control. Moreover, the combination of SSe with *α*-TOS inhibited VEGF release with the same efficacy as *α*-TOS (Figures 6(a) and 6(b)).

### 3.9. *α*-TOS, SSe, and Their Combinations Decrease Tumor Volume In Vivo

**Figure 7:**
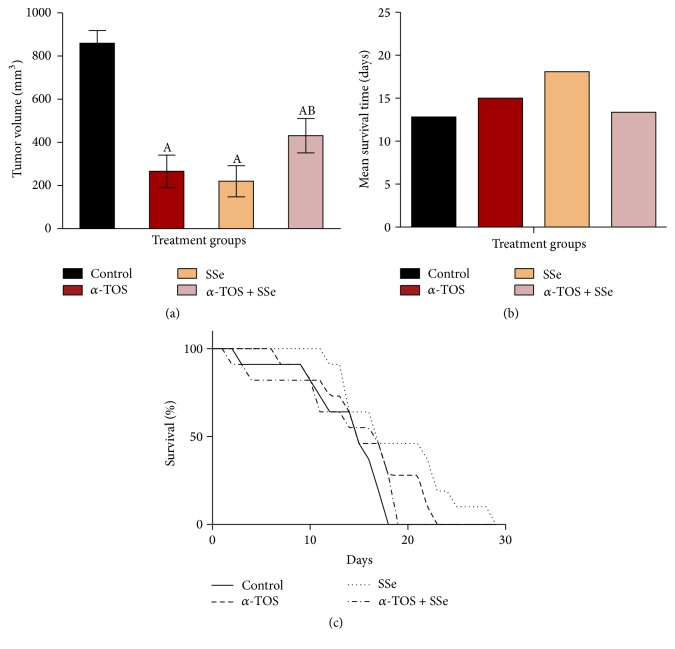
In vivo effects of administration of *α*-TOS (300 mg/kg), SSe (1 mg/kg), and their combination on (a) tumor volume of solid Ehrlich carcinoma-bearing mice, (b) mean, and (c) percent survival of EAC. Results of tumor volume are expressed as means ± SD. “A” significantly different from the respective control at *P* < 0.05; “B” significantly different from respective *α*-TOS at *P* < 0.05.

The volume of solid tumor in untreated control reached a size of 860 mm^3^ 7 days from tumor inoculation. However, it reached 266 mm^3^ and 220 mm^3^ 7 days from tumor inoculation following treatment with *α*-TOS and SSe, respectively, while the combined treatment resulted in a tumor volume of 431 mm^3^ which is significantly larger than *α*-TOS only (Figure 7(a)).

### 3.10. SSe Abrogates the Oncolytic Activity of *α*-TOS

Regarding the percent survival of mice, on day 18, none of the control tumor-bearing mice were alive, on day 23, none of the *α*-TOS-treated mice were alive, and on day 29 none of the SSe-treated mice were alive. Concerning the combination, on day 22, none of the mice were alive. Also, *α*-TOS, SSe, and their combination increased the life span of mice by 17.2, 41.4, and 3.9%, respectively (Table 2 and Figures 7(b) and 7(c)).

## 4. Discussion

In the present study, *α*-TOS inhibited the proliferation of MCF7 cells, with an early significant increase in MDA. Similar studies reported antitumor activity for *α*-TOS on different cancer cell lines, including prostate cancer [26], gastric cancer [27], pancreatic cancer [4], resistant mesothelioma [28], and HER2 overexpressing breast cancer cell line [29]. This cytotoxicity was convoyed by an early buildup of ROS, upon exposure to *α*-TOS in Jurkat cells [30], breast cancer cells [29], melanoma cells [31], prostate cells [32], and non-small cell lung cancer cells [33]. As a member of the mitocans, *α*-TOS disrupts the mitochondrial membrane potential causing the generation of ROS resulting in apoptosis [34]. *α*-TOS induced activation of caspases 7 and 9 and increased activity of caspase 3 without changes in the expression of antiapoptotic protein levels (Bcl-2 and Mcl-1) of MCF7 cells in our study. However, Gu et al. [35] found dramatic decrease in Bcl-2 protein level at 6 hours followed by a slight recovery at 12 hours suggesting metabolic degradation of *α*-TOS upon prolonged incubation. Kang et al. [33] found that cytotoxicity induced by *α*-TOS was cell type dependent. It was abrogated by prior addition of antioxidants, explaining the role of ROS in *α*-TOS-induced apoptosis. However, it was described that incubation of glioblastoma cancer cells with *α*-TOS resulted in apoptosis with negligible effects on ROS. Moreover, the presence of an antioxidant did not alter the rate of cell death. Moreover, ROS have been copiously reported as early inducers of autophagy upon nutrient deprivation. In addition, it is an evolutionarily conserved catabolic process, responsible for the routine degradation of bulk dysfunctional proteins and organelles [36]. Autophagy plays a protective role in response to a majority of anticancer drugs and in the pathogenesis process [36]. In the current study, we found that *α*-TOS produced early induction of autophagy manifested by increased beclin-1 protein level and an early increase in the expression of LC3B protein, responsible for the completion of the autophagosome formation, which recovered after prolonged incubation to control value. Likewise, Neuzil et al. [37] reported early or initiating lysosomal destabilization event in apoptosis induced by *α*-TOS that precedes both caspases activation and phosphatidyl serine externalization. They suggest that the key player in apoptosis was cathepsin D and cathepsin D-deficient cells showed lower caspase 3 activity and resist apoptosis.

Complementary therapies including dietary supplements, herbs, and vitamins play a major role in cancer prevention if utilized properly; they can change the course of cancer progression. Many dietary factors affect the rate of growth of cancerous tumors and specific dietary interventions may potentially reverse tumor progression. Results of this study showed inhibition of growth of MCF7 cells exposed to SSe. Low concentration of SSe significantly increased level of antioxidant glutathione, while higher concentration produced significant increase in ROS. Similarly, the anticancer activity of SSe was reported in previous studies on osteosarcoma [38], malignant mesothelioma [12], prostate cancer [39], and lung cancer cell lines [40]. The effect of SSe on ROS in different cell lines is controversial. In harmony with our data, Chatzakos et al. [41] and Fu et al. [42] reported that low concentrations of SSe can regulate cellular redox levels, resist peroxidation, and protect against cancer, while higher concentrations exert an oxidative stress resulting in ROS-mediated apoptosis. Sarada et al. [43] reported that addition of SSe to neuroblastoma cells in culture prior to hypoxia-induced ROS decreased the hypoxia-induced cell death. It was suggested that the resultant activation of the caspases cascade by SSe may be ROS independent and an alternative pathway might be considered [38, 42, 44]. In addition, Park et al. [44] found that treatment of human lung carcinoma cell line with SSe resulted in an early modulation of the extrinsic apoptotic pathway, represented by an upregulation in the expression of Fas and death receptor, which was coupled with decreased expression of pro-Bid suggesting that truncated Bid might have served to connect both extrinsic and intrinsic apoptotic pathways. This was confirmed by suppression in the expression of procaspases 8, 9, and 3 by SSe, confirming activation of caspases by SSe via both apoptotic pathways. On the other hand, SSe was declared by others as a prooxidant catalyst [45–47]. Moreover, in this study SSe also activated autophagy by an increased expression of protein levels and presence of AVOs in the cytoplasm of SSe-treated cells. Available evidence suggests that SSe may stimulate or inhibit autophagy by diverse mechanisms including superoxide-targeting mitochondria [48] and mTOR signaling [49] or via beclin-1 transcriptional inhibition linked to heat shock protein 90 and nuclear factor kappa B [50]. The outcome of the combined effect of *α*-TOS with SSe in this study was found to be antagonistic with decrease in caspases activation compared to *α*-TOS alone. SSe did not affect the cellular uptake of *α*-TOS from the culture medium excluding that the decrease in cytotoxicity may be due to decrease in drug concentration. The antagonistic effect of SSe may contribute to inhibition of ROS induced by *α*-TOS and increase of antioxidant (glutathione) level by SSe which counteract the oxidative stress induced by *α*-TOS. It was declared that antioxidants could partially inhibit *α*-TOS-induced cell death in ROS dependent cells and that caspase-dependent apoptotic pathway is always involved in *α*-TOS-induced cell death, regardless of ROS dependence of cells. Moreover, it was found that inhibition of ROS generation did not inhibit the activation of caspases and that another mediator that activates caspase-dependent apoptosis may be present without relation to ROS generation in response to *α*-TOS [33]. In this study, although SSe inhibited ROS levels and apoptosis in the combination regimen, the induction of autophagy was sustained which maybe contributed to the effect of SSe. Available evidence suggests that SSe may stimulate or inhibit autophagy by diverse mechanisms [49]. In addition evidence shows that autophagy in SSe-treated cells represents a concurrent process which reduces apoptosis rate [49]. To elucidate the relationship between both apoptosis and autophagy in our study, we treated cells with an autophagy inhibitor CQ. We noticed a dramatic decline in survival of cells treated with SSe and CQ was observed suggesting a prosurvival mechanism. Previous studies by Ren et al. [49], Park et al. [44], and Králová et al. [51] also confirmed a prosurvival role for SSe-induced autophagy. Although it is known that ROS can trigger autophagic cell death, other studies showed that ROS functions as a survival mechanism via induction of cytoprotective autophagy in several cancer cells [52, 53]. These types of cells induced autophagy as a means of adaption to stressful conditions.

Our in vitro findings were confirmed in vivo showing that *α*-TOS and SSe alone were capable of reducing the volume of solid tumor. However, the coadministration of *α*-TOS and SSe resulted in significantly larger tumor volume compared to *α*-TOS treatment group. Several reports have also documented the in vivo antitumor effects of *α*-TOS in lung cancer tumor [5] and colon cancer tumor [54], as well as reducing the vitality of EAC-bearing mice [55]. In addition SSe also reduced the volume of solid tumor in mice as shown by us and others [56]. In conclusion, using complementary therapies with conventional cancer therapy for symptom management and enhancement of quality of life should be intensively studied. SSe in low concentration may protect MCF7 cells from cell death induced by *α*-TOS via inhibiting apoptosis and induction of prosurvival autophagy.

## Supplementary Material

Cytotoxic effect of different concentrations of SSe (2 - 10 μM) and α-TOS (20 – 100 μM) and their combination with 2 μM and 10 μM SSe on different cancer cell lines(Breast adenocarcinoma MCF7, cervical adenocarcinoma HeLa, lung carcinoma A549, mammary gland ductal carcinoma T47D, prostate adenocarcinoma PC3, hepatocellular carcinoma HepG2, and colorectal carcinoma HCT-116 cell lines) after 48 hours of incubation.

## Figures and Tables

**Table 1:** Screening of cytotoxic effect of SSe (2–10 *μ*M) and *α*-TOS (20–100 *μ*M) and their combination with 2 *μ*M and 10 *μ*M SSe and the resultant combination indices after 48 hours on MCF7 cells. The results are obtained from 5 independent experiments performed in triplicate.

Human cancer cell lines	The 50% inhibitory concentration				Combination index (CI)	
	TOS (*μ*M)	SSe (*μ*M)	TOS + 2 *μ*M SSe	TOS + 10 *μ*M SSe	TOS + 2 *μ*M SSe	TOS + 10 *μ*M SSe
HeLa	>100	>10	>100	>100	1.05	2.37
A549	82	>10	90.5	>100	1.86	2.19
T47D	>100	>10	>100	>100	1.42	2.24
PC3	>100	>10	>100	>100	1.50	2.38
HepG2	83.3	>10	93.2	98.2	2.08	3.07
HCT-116	76.8	>10	89.8	91.3	1.25	2.22
MCF7	57.5	5.45	65.5	12.5	1.50	2.09

**Table 2:** Effect of administration of *α*-TOS (300 mg/kg), SSe (1 mg/kg), and their combination on the mean survival time (MST) and percentage change in life span (CLS) in EAC-bearing mice.

Treatment (mg/kg)	MST ± SD	CLS (%)
Control	12.8 ± 4.5	—
*α*-TOS	15.0 ± 5.2	17.2
SSe	18.1 ± 5.6	41.4
*α*-TOS + SSe	13.4 ± 6.7	3.9
