# Supplementary material for: The Combination of α-Tocopheryl Succinate and Sodium Selenite on Breast Cancer: A Merit or a Demerit?
Source: Oxid Med Cell Longev. 2016 Mar 29;2016:4741694. doi: 10.1155/2016/4741694 (PMC4834195; doi:10.1155/2016/4741694)
Supplement: Supplementary file 1 [file 4741694.f1.docx]

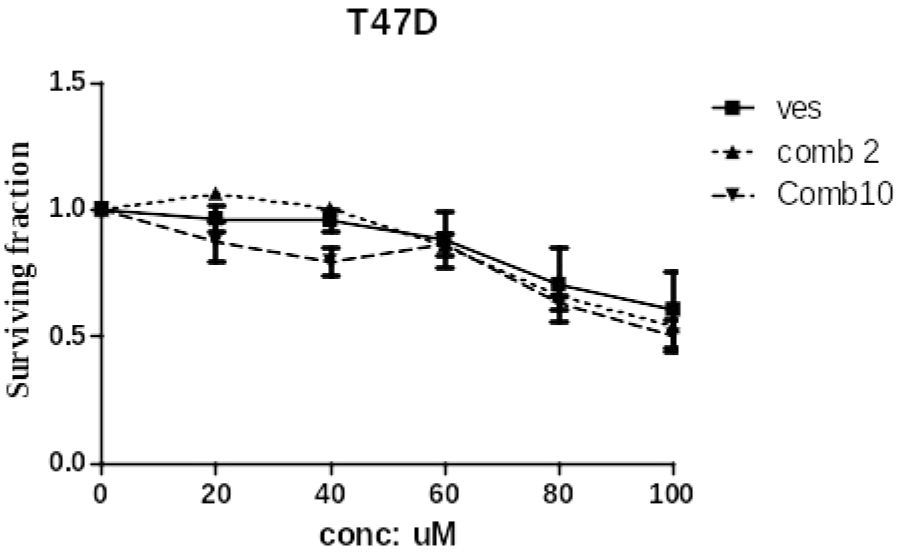
T47D
Surviving fraction
conc: uM
ves
comb 2
Comb10
1.5
1.0
0.5
0.0
0
20
40
60
80
100

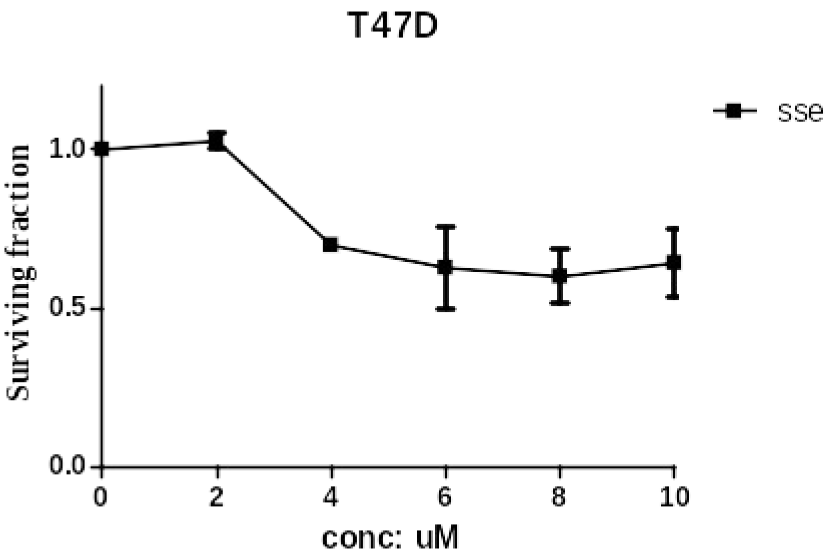
T47D
Surviving fraction
conc: uM
sse
1.0
0.5
0.0
0
2
4
6
8
10

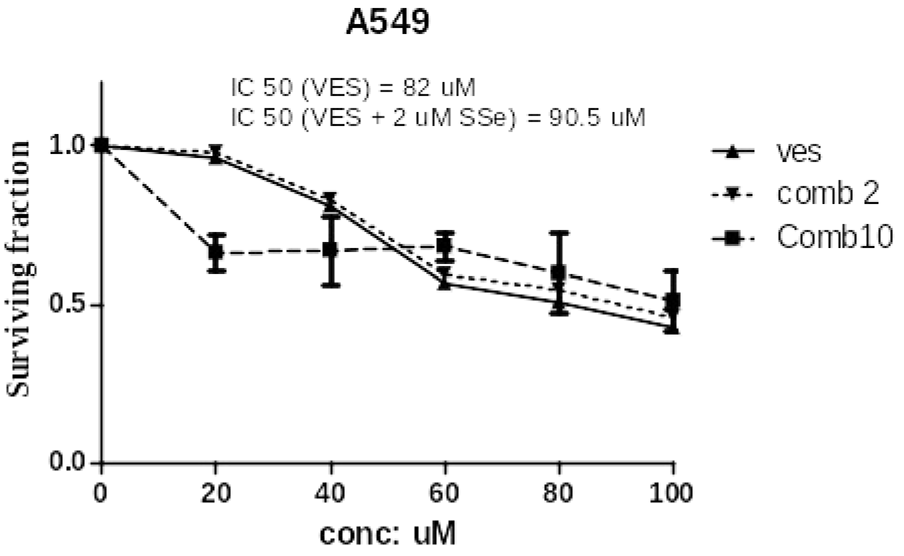
A549
IC 50 (VES) = 82 uM
IC 50 (VES + 2 uM SSe) = 90.5 uM
Surviving fraction
1.0
0.5
0.0
0
20
40
60
80
100
conc: uM
ves
comb 2
Comb10

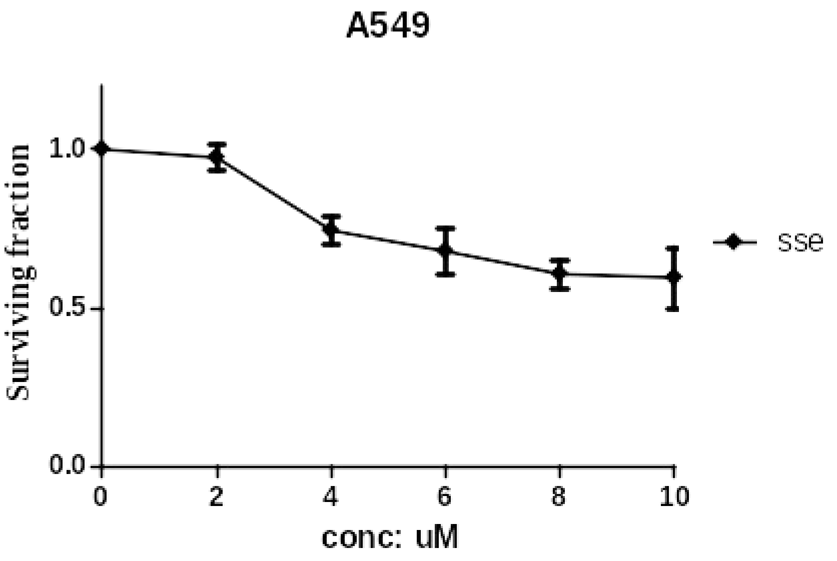
A549
Surviving fraction
1.0
0.5
0.0
0
2
4
6
8
10
conc: uM
sse

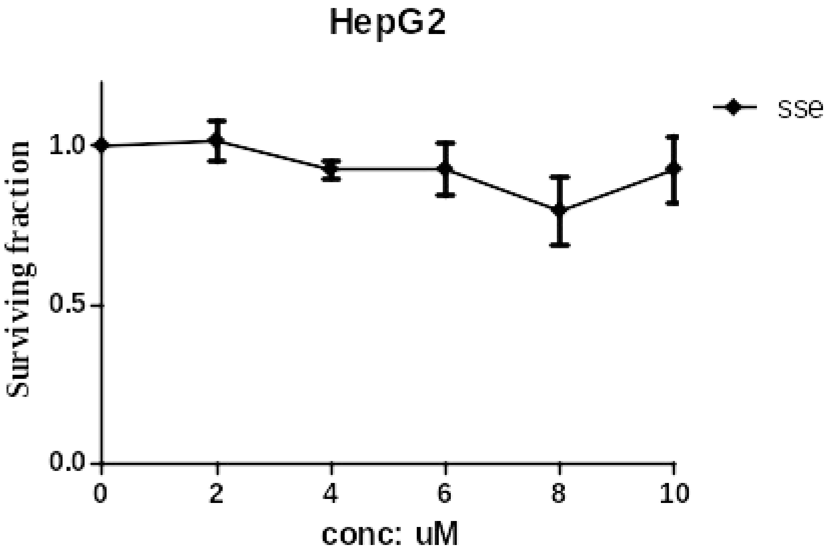
HepG2
sse
Surviving fraction
1.0
0.5
0.0
0
2
4
6
8
10
conc: uM

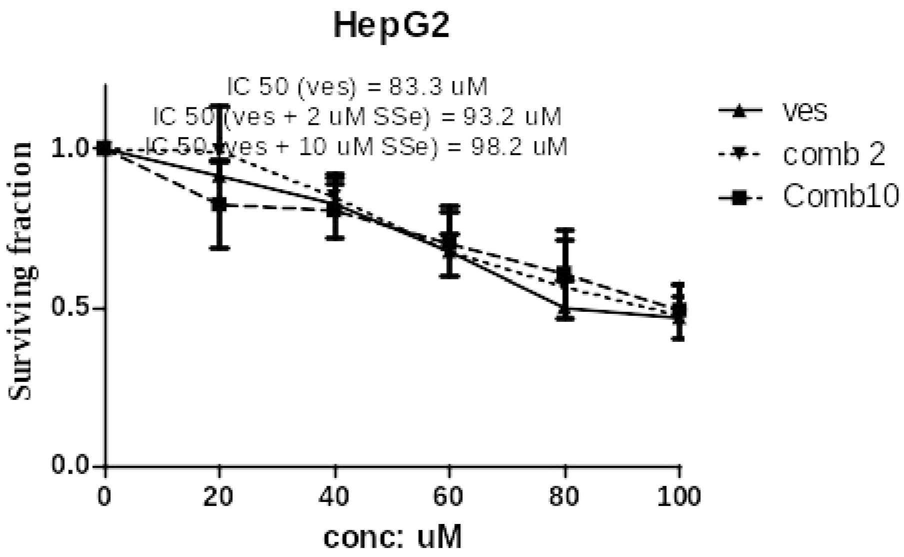
HepG2
IC 50 (ves) = 83.3 uM
IC 50 (ves + 2 uM SSe) = 93.2 uM
IC 50 (ves + 10 uM SSe) = 98.2 uM
ves
comb 2
Comb10
Surviving fraction
1.0
0.5
0.0
0
20
40
60
80
100
conc: uM

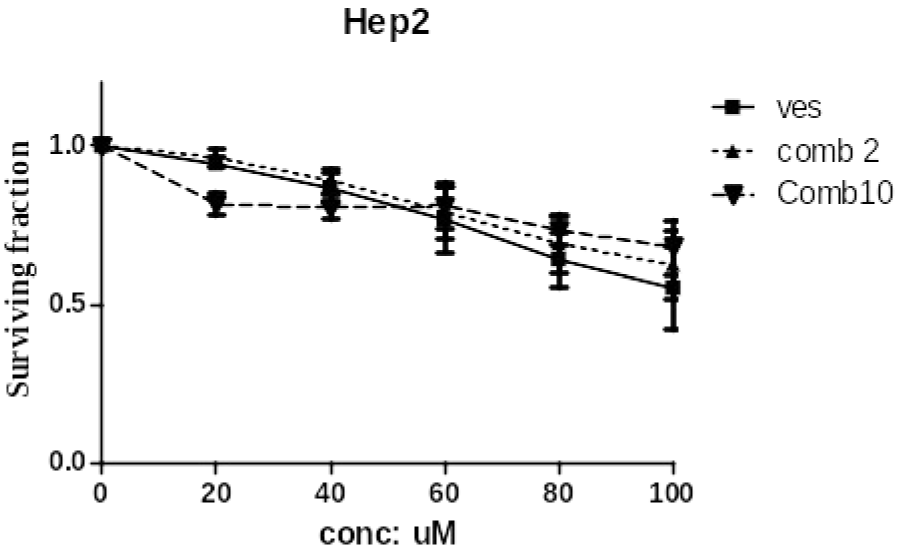
Hep2
Surviving fraction
1.0
0.5
0.0
0
20
40
60
80
100
conc: uM
ves
comb 2
Comb10

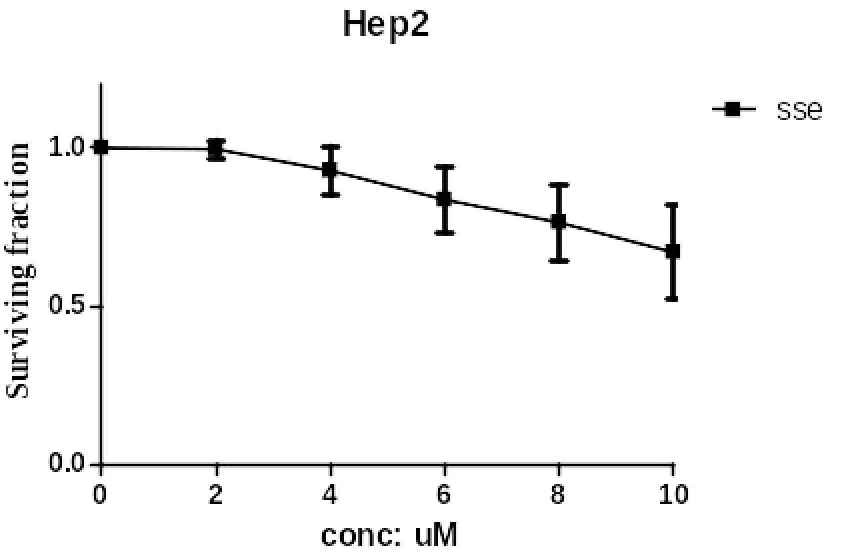
Hep2
Surviving fraction
1.0
0.5
0.0
0
2
4
6
8
10
conc: uM
sse

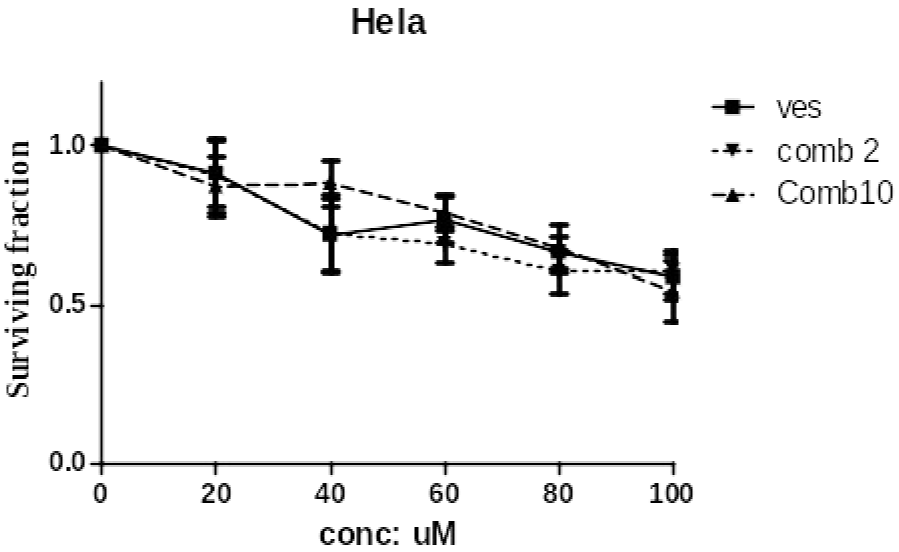
Hela
Surviving fraction
1.0
0.5
0.0
0
20
40
60
80
100
conc: uM
ves
comb 2
Comb10

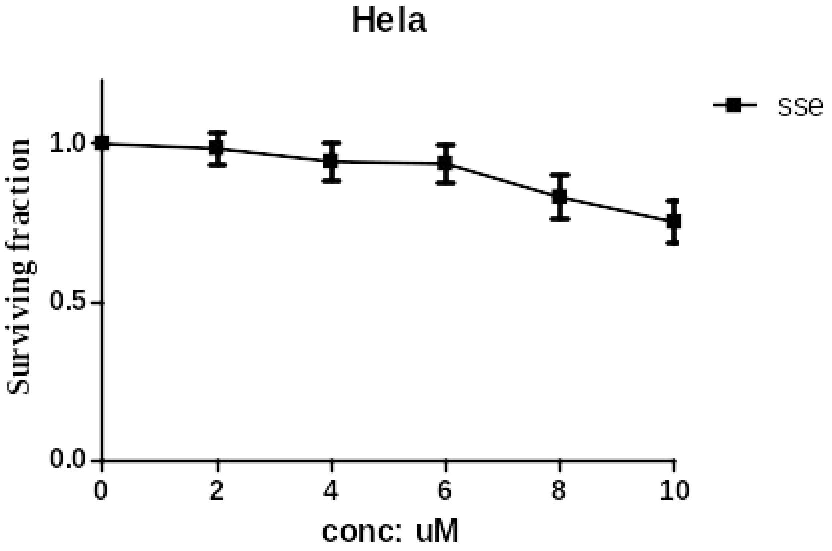
Hela
Surviving fraction
1.0
0.5
0.0
0
2
4
6
8
10
conc: uM
sse
